# P-1553. Exploring the Role of Capsule in Innate Immune Interactions of Multidrug-Resistant Klebsiella pneumoniae

**DOI:** 10.1093/ofid/ofaf695.1733

**Published:** 2026-01-11

**Authors:** Nathalie Chen, Margaret Cassady, Emma Mills, Shekina Gonzalez-Ferrer, Marrisa P Griffifth, Lora Pless, Lee Harrison, William Bain, Daria Van Tyne

**Affiliations:** University of Pittsburgh, Pittsburgh, PA; University of Pittsburgh, Pittsburgh, PA; University of Pittsburgh, Pittsburgh, PA; University of California, Los Angeles, Pittsburgh, Pennsylvania; University of Pittsburgh, Pittsburgh, PA; University of Pittsburgh, Pittsburgh, PA; University of Pittsburgh, Pittsburgh, PA; University of Pittsburgh, Pittsburgh, PA; University of Pittsburgh School of Medicine, Pittsburgh, Pennsylvania

## Abstract

**Background:**

*Klebsiella pneumoniae* (KP) belonging to multi-locus sequence type 258 (ST258) is a frequent cause of hospital-associated outbreaks. KP ST258 isolates display extensive multidrug-resistance; however, this feature alone does not explain the global dissemination and pathogenic success of this lineage. KP ST258 consists of two genetically distinct clades, Clade 1 and Clade 2. While bacteria belonging to both clades are isolated from clinical infections, Clade 2 is isolated more frequently compared to Clade 1.

**Methods:**

To investigate drivers of this difference in distribution, we leveraged a collection of 50 sequenced clinical KP ST258 isolates (N=13 Clade 1, N=37 Clade 2) to genotypically and phenotypically assess the two clades.

**Results:**

A primary difference between the two clades is the polysaccharide capsule, which is a critical virulence factor for KP. Analysis of the Clade 1 capsule showed that it consists of glucose/glucuronic acid (86.1%) and galactose (13.9%), while the Clade 2 capsule consists of rhamnose (66.2%) and galactose/galacturonic acid (33.8%). We found that Clade 1 isolates were more frequently isolated from urine specimens, while Clade 2 isolates were more frequently associated with respiratory and blood specimens. On average, Clade 1 isolates had larger genomes than Clade 2 isolates (5.66 Mb vs 5.57 Mb, respectively), and they also had more plasmid replicon genes (4.7 vs 1.9 genes, respectively), and more antibiotic resistance genes (9.6 vs 6.8 genes, respectively). These genome differences would suggest that Clade 1 isolates might be more successful pathogens than Clade 2 isolates, however we found that mice intratracheally infected with a Clade 2 isolate died more rapidly than Clade 1-infected mice. We also found that Clade 2 isolates were more resistant to killing by human serum, despite binding more complement C3 than Clade 1 isolates. Furthermore, Clade 2 isolates produced more C5a upon exposure to human serum than Clade 1 isolates.

**Conclusion:**

Taken together, these data suggest that KP ST258 Clade 2 is more pathogenic compared to Clade 1, perhaps because of its capsule. Future work examining these two clades may reveal targeted therapeutic strategies to improve the prevention and treatment of multidrug-resistant KP infections.

**Disclosures:**

Lora Pless, PhD, Astra Zeneca - Icosavax: Grant/Research Support Lee Harrison, MD, GSK: Advisor/Consultant|Merck: Board Member|Pfizer: Advisor/Consultant|Sanofi: Advisor/Consultant

